# Metaproteomics-based stable isotope fingerprinting links intestinal bacteria to their carbon source and captures diet-induced substrate switching

**DOI:** 10.1093/ismejo/wraf127

**Published:** 2025-06-24

**Authors:** Angie Mordant, J Alfredo Blakeley-Ruiz, Manuel Kleiner

**Affiliations:** Department of Plant and Microbial Biology, North Carolina State University, Raleigh, NC 27695, United States; UNC Metabolomics and Proteomics Core Facility, Department of Pharmacology, University of North Carolina at Chapel Hill, NC, United States; Department of Plant and Microbial Biology, North Carolina State University, Raleigh, NC 27695, United States; Department of Plant and Microbial Biology, North Carolina State University, Raleigh, NC 27695, United States

**Keywords:** gut microbiota, protein-based stable isotope fingerprinting, metaproteomics, microbiome

## Abstract

Diet has strong impacts on the composition and function of the gut microbiota with implications for host health. Therefore, it is critical to identify the dietary components that support growth of specific microorganisms *in vivo*. We used protein-based stable isotope fingerprinting (Protein-SIF) to link microbial species in gut microbiota to their carbon sources by measuring each microorganism’s natural ^13^C content (δ^13^C) and matching it to the ^13^C content of available substrates. We fed gnotobiotic mice, inoculated with a 13 member microbiota, diets in which the ^13^C content of all components was known. We varied the source of protein, fiber, or fat to observe ^13^C signature changes in microbial consumers of these substrates. We observed significant changes in the δ^13^C values and abundances of specific microbiota species, as well as host proteins, in response to changes in ^13^C signature or type of protein, fiber, and fat sources. Using this approach we were able to show that upon switching dietary source of protein, fiber, or fat (i) some microbial species continued to obtain their carbon from the same dietary component (e.g. protein); (ii) some species switched their main substrate type (e.g. from protein to carbohydrates); and (iii) some species might derive their carbon through foraging on host compounds. Our results demonstrate that Protein-SIF can be used to identify the dietary-derived substrates assimilated into proteins by microorganisms in the intestinal tract; this approach holds promise for the analysis of microbiome substrate usage in humans without the need of substrate labeling.

## Introduction

Interactions between the intestinal microbiota and diet play key roles in health and disease [1]. For example, short-chain fatty acids derived from dietary fiber and protein fermentation by the gut microbiota play a role in mucus layer formation [2, 3], are anti-inflammatory [4], and are one of the primary nutrient sources for host colonocytes [5], whereas fermentation of specific amino acids derived from proteins also produce toxins like putrescine, ammonia, and hydrogen sulfide which can be detrimental to host health [6, 7]. Oftentimes the key assumption made in studies investigating the effects of diet on the gut microbiota is that the microorganisms that respond to a diet consume specific dietary components and that this drives changes in their abundance [8–10]. Correlations between diet changes and taxon abundances, however, could also be due to other causes, such as the effect of antimicrobial factors in foods [11], cross-feeding on byproducts from other species that consume dietary components [12], or switching between use of dietary substrates and host foraging [13, 14]. Because microbiota response to diet can have major health consequences, tools to determine the use of particular dietary components by specific microbiota members are urgently needed.

Specific diet-derived substrates consumed by intestinal microorganisms have been inferred using a variety of existing approaches. These approaches include using individual microorganisms or defined communities in gnotobiotic animals in combination with gene expression analyses and *in vitro* growth of bacteria on dietary components [13, 15]; strains in which genes for use of specific substrates were knocked out [16] or knocked in [17]; or stable isotope probing (SIP) using labeled substrates and measuring the ratio of stable carbon isotope (i.e. expressed as δ^13^C) in cellular components [18]. δ^13^C is the ratio of ^13^C to ^12^C in an organism calibrated by the ^13^C to ^12^C ratio of a standard and is defined as


\begin{equation*} \delta^{13}\text{C} = \left( \frac{\left(^{13}\text{C}/^{12}\text{C}\right)_{\text{sample}}}{\left(^{13}\text{C}/^{12}\text{C}\right)_{\text{standard}}} - 1 \right) \times 1000 \end{equation*}

The δ^13^C of cellular components is a particularly powerful method because it is the only method that can directly identify the nutrient sources of different organisms by comparing the isotopic ratio of an organism’s cellular components (e.g. protein, DNA, lipids) with the isotopic ratio(s) of the nutrient source(s) the organism used to build those components [19–23]. Already, insights into nutrient flows in gut microbiota have been obtained by SIP approaches, where labeled compounds are injected into the bloodstream or provided as dietary components to the host and then incorporation of the labeled compound is determined by observing changes in the isotopic signatures of DNA, proteins, or metabolites from the host or microbiota. For example, ^15^N- and ^13^C-labeled threonine injected into the bloodstream of mice revealed variable incorporation of these labels, presumably from proteins secreted into the gut, by microbial taxa in the intestine using a high-resolution secondary ion mass spectrometry (NanoSIMS) approach. This revealed variable preferences for host versus dietary proteins across different microbial taxa [24, 25]. In addition, oral or injected provision of a wide array of different labeled proteins and metabolites to healthy mice fed a standard chow diet revealed the nutrient sources for different microorganisms and their associated metabolites [18]. For example, short-chain fatty acids were shown to primarily be produced by the fermentation of fiber as opposed to amino acids, and different bacterial taxa were shown to favor dietary protein, urea, or host protein as their source of nitrogen, but specifically did not use circulating amino acids. Although these SIP studies provide tremendous insight into the steady state preferences of bacterial taxa for specific substrates, they are limited by the need to deliver labeled compounds to the intestine, which limits SIP’s future useability in humans, but also makes it difficult to explore the nuance of preference for different sources of macronutrients (e.g. different sources of fiber, protein, and fat) by bacteria in the intestine.

Mass spectrometry based shotgun metaproteomics on its own can provide insight into bacterial preferences for different macronutrients because it is able to capture how gene expression in specific microorganisms responds to changes in host diet. For example, in gnotobiotic mice it was shown that switching fiber source changes the expression of genomic regions encoding glycoside hydrolases in *Bacteroides* species [26] and more recently we showed that *Bacteroides thetaiotaomicron* gene expression changes in the presence of different dietary protein sources [27]. This approach tells us how the nutrient sources are affecting the gene expression of the specific organisms and gives us some hints as to what nutrients the microorganisms are consuming, but fails to provide direct evidence for which nutrient sources the bacteria are actually incorporating.

We recently developed a metaproteomics method (protein stable isotope fingerprinting [protein-SIF]) for measuring the natural stable carbon isotope fingerprints (δ^13^C) of specific species in a microbial community, which could be used in tandem with metaproteomic gene expression analysis to determine the carbon substrate of intestinal bacteria. Living organisms carry their own distinct carbon isotopic signature based on their carbon source (substrate/food) and this signature, or stable isotope fingerprint (SIF), can be used to infer the organism’s carbon source(s), as has been done in field ecology studies [19]. Our protein-SIF approach for microbial communities was validated in vitro and tested in a case study using a gutless worm, which revealed new insights into the carbon sources of the worm’s microbial symbionts [20]. Herein we describe our application of protein-SIF to gnotobiotic mice, colonized with a defined bacterial community, that were fed a series of controlled diets that varied in the type of available protein, fiber, or fat. We separately measured the δ^13^C signature of each dietary component so it would be possible to link specific dietary components to specific microbial species and to the host through changes in their δ^13^C. Our results suggest that protein-SIF provides direct evidence for substrate preferences of specific members of a microbial community without the need for labeled compounds.

## Materials and methods

### Overall experimental design

**Figure 1:**
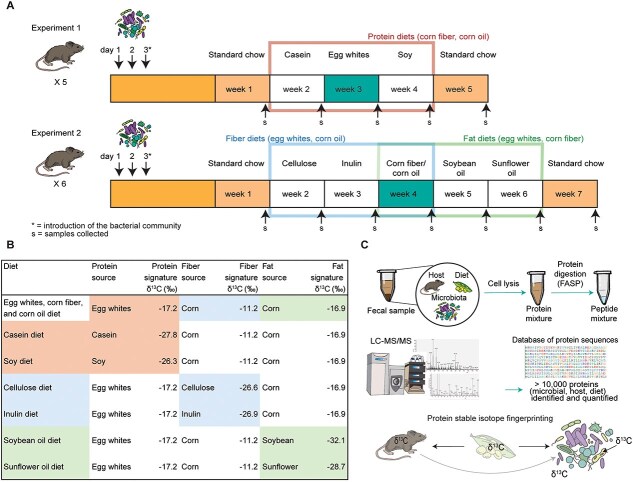
Overview of experimental design and procedure. A. Timeline and design of the experiment. The diet in the blue weeks (week 3 in Exp1 and week 4 in Experiment 2) was the same exact diet. B. Natural carbon isotopic signatures (δ^13^C values) of the macronutrients used in the diets. Signatures of dietary components were averaged between two replicates measured by EA-IRMS (measurement uncertainties of ±0.42‰ or less; ± 0.13‰ averaged uncertainty). C. Overview of the metaproteomics protein-SIF approach.

We conducted two experiments to investigate the assimilation of dietary macronutrients by intestinal bacteria in gnotobiotic mice colonized with a 13 member defined community [13] (Table 1; Fig. 1A). In Experiment 1 (n = 5) we fed gnotobiotic mice defined diets differing only in the source of dietary protein (egg white, casein, or soy protein). In Experiment 2 (n = 6) we fed gnotobiotic mice defined diets that differed either only in their source of dietary fiber (cellulose, inulin, or corn fiber) or in their source of dietary fat (corn oil, soybean oil, or sunflower oil). All of these nutrients had distinct natural isotopic signatures (Fig. 1B). Each diet was fed for 1 week and a fecal sample was taken from each mouse at the end of the week. Fecal samples were stored in a nucleic acid preservation (NAP) buffer upon collection and then frozen at −80°C within hours of collection [28]. We also collected baseline samples prior to transitioning to the defined diets when the mice had been eating a standard chow diet (Lab diet 5010) for 21 days after colonization and again 1 week after returning to the standard chow at the end of each experiment. We measured all the samples using a protein-SIF metaproteomic approach (Fig. 1C) [20].

**Table 1:** Organisms/strains of the defined community.

Species	Strain	Original Source (before laboratory of Dr. Eric Martens)	Phylum
*Bacteroides ovatus*	1896, Type strain	DSMZ	Bacteroidota
*Bacteroides uniformis*	8492	ATCC	Bacteroidota
*Bacteroides thetaiotaomicron*	2079, Type strain	DSMZ	Bacteroidota
*Bacteroides caccae*	19 024, Type strain (ATCC43185)	DSMZ	Bacteroidota
*Barnesiella intestinihominis*	YIT11860 (JCM15079)	ATCC	Bacteroidota
*Roseburia intestinalis*	14 610, Type strain L1–82	DSMZ	Bacillota
*Eubacterium rectale*	17 629, A1–86	DSMZ	Bacillota
*Faecalibacterium prausnitzii*	17 677, A2–165	DSMZ	Bacillota
*Marvinbryantia formatexigens*	14 469, Type strain I-52	DSMZ	Bacillota
*Clostridium symbiosum*	934, Type strain, designation 2	DSMZ	Bacillota
*Collinsella aerofaciens*	3979, Type strain	DSMZ	Actinomycetota
*Escherichia coli*	HS	ATCC	Pseudomonadota
*Akkermansia muciniphila*	22 959, Type strain, Muc	DSMZ	Verrucomicrobiota

### Gnotobiotic models

We used 11 germ-free C57BL/J6 mice (five in Experiment 1 and six in Experiment 2, all females). The NCSU Gnotobiotic core supplied and housed the mice. The mice were housed in groups of three throughout the experiment, except for one cage, which had two mice. All animal experiments followed protocols approved by the Institutional Animal Care and Use Committee (IACUC) of North Carolina State University.

The germ-free mice were colonized with the defined community (Table 1) at 9–11 weeks of age with freshly prepared bacterial inocula. Bacteria were grown in individual cultures in their respective media [13]. The cultures were grown anaerobically in Hungate tubes. The cultures were incubated at 37°C for 1–2 days depending on the strain until they reached optical densities (OD, absorbance at 600 nm) ranging from ~0.2 to >2. Bacterial cultures were mixed in equal volumes. We gavaged each mouse with 200 μl of the bacterial mixture for three consecutive days.

### Design of diets with known isotopic signatures

We designed seven diets for this study based on the AIN93G [29] diet with minor modifications (Suppl. Table S1). All seven diets contained the same amounts of corn starch, maltodextrin, and sucrose. We included three groups of diets: “protein diets", “fiber diets", and “fat diets”. In each experiment, we compared three sources of protein, fiber, or fat. Every diet had one ingredient with a distinct isotopic signature from the other components in the diet. One ingredient with a distinct isotopic signature was an important experimental design feature as it allowed us to clearly point to the cause of a change in the δ^13^C of a specific microorganism. To design the diets, we measured the natural isotopic signatures of purified ingredients obtained from Envigo Teklad and Amazon. We weighed the ingredients into tin capsules (~ 1 mg per sample) and analyzed them by Elemental Analysis Isotope Ratio Mass Spectrometry (EA-IRMS) (Fig. 1B). The ingredients were compared to a Vienna Pee Dee Belemnite standard using area under the curve calculations to determine the carbon isotope ratios. We analyzed each purified ingredient in duplicate because IRMS measurements are highly robust (Suppl. Table S2). All defined diets were purchased from Envigo Teklad. All defined diets were sterilized by gamma irradiation and vacuum packaged.

### Protein extraction and peptide preparation

Proteins for metaproteomics were extracted from samples of the seven diets (Suppl. Table S1), purified casein and purified egg white solids (to serve as a standard; see below), and feces from the 11 experimental mice at timepoints indicated in Fig. 1A. We extracted the diets and purified ingredient samples in triplicate. Each replicate consisted of 100 mg of diet that we powdered down from diet pellets or 100 mg of a purified ingredient. For the fecal microbiome samples, we extracted one fecal pellet per mouse per diet. If applicable, we removed the NAP buffer from the samples by centrifugation at 21000 × g for 5 min. We suspended the samples in 400 μl of SDT lysis buffer [4% (w/v) SDS, 100 mM Tris–HCl pH 7.6, 0.1 M DTT]. Cells were lysed by bead-beating in lysing matrix E tubes (MP Biomedicals) with a Bead Ruptor Elite (Omni International) for five cycles of 45 sec at 6.45 m/s with 1 min dwell time between cycles, followed by heating at 95°C for 10 min. Bead-beating was applied to the diet and the fecal samples but not the purified protein powders, which were heated at 95°C for 10 min. The lysates were centrifuged for 5 min at 21000 × g to remove cell debris. We prepared peptides according to the filter-aided sample preparation protocol [30]. All centrifugations were performed at 14000 × g. Samples were loaded twice onto 10 kDa MWCO 500 μl centrifugal filters (VWR International) by combining 60 μl of lysate with 400 μl of Urea solution (8 M urea in 0.1 M Tris/HCl pH 8.5) and centrifuging for 30 min. Filters were washed twice by applying 200 μl of urea solution followed by 40 min of centrifugation; 100 μl IAA solution (0.05 M iodoacetamide in urea solution) was then added to filters for a 20 min incubation followed by centrifugation for 20 min. Filters were washed three more times by adding 100 μl of ABC (50 mM ammonium bicarbonate) followed by centrifugation for 20 min. Tryptic digestion was performed by adding 0.85 μg of MS grade trypsin (Thermo Scientific Pierce, Rockford, IL, USA) in 40 μl of ABC to the filters and incubating for 16 hours at 37°C. The tryptic peptides were eluted by adding 50 μl of 0.5 M NaCl and centrifuging for 20 min. Peptide concentrations were determined with the Pierce Micro BCA assay (ThermoFisher Scientific) following the manufacturer's instructions.

We mixed the peptides from the purified casein and the purified egg white solids in equal concentrations to create a casein and egg sample that was later used as a calibration standard for the protein-SIF approach.

### Liquid chromatography and mass spectrometry

Samples were analyzed by reverse phase chromatography followed by tandem mass spectrometry (LC-MS/MS) as described previously [28]. The samples were blocked and randomized to control for batch effects. Alongside the fecal microbiome samples we included, at the beginning and end of the run sequence, two types of protein-SIF standards: the casein/soy protein standard mentioned above and peptides from human hair that were measured with EA-IRMS. Every sample was run as four consecutive technical replicates to increase the number of peptides available for protein-SIF. Only the human hair protein-SIF standard was run as a single replicate as it contained enough peptides. For each sample replicate, 600 ng of tryptic peptides were loaded with an UltiMate 3000 RSLCnano Liquid Chromatograph (ThermoFisher Scientific) in loading solvent A (2% acetonitrile, 0.05% trifluoroacetic acid) onto a 5 mm, 300 μm ID C18 Acclaim® PepMap100 pre-column and desalted (ThermoFisher Scientific). Peptides were then separated on a 75 cm × 75 μm analytical EASY-Spray column packed with PepMap RSLC C18, 2 μm material (ThermoFisher Scientific) heated to 60°C via the integrated column heater at a flow rate of 300 nl min^−1^ using a 140 min gradient.

The analytical column was connected to a Q Exactive HF hybrid quadrupole-Orbitrap mass spectrometer (ThermoFisher Scientific) via an Easy-Spray source. MS1 spectra were acquired by performing a full MS scan at a resolution of 60 000 on a 380 to 1600 m/z window. MS2 spectra were acquired using a data-dependent approach by selecting for fragmentation the 15 most abundant ions from the precursor MS1 spectra. A normalized collision energy of 25 was applied in the HCD cell to generate the peptide fragments for MS2 spectra. Other settings of the data-dependent acquisition included: a maximum injection time of 100 ms, a dynamic exclusion of 25 sec, and exclusion of ions of +1 charge state from fragmentation.

### Protein identification database

We constructed a protein sequence database for identifying proteins from the components of the fecal samples (i.e. the microbiota, the host, the dietary components, and potential contaminants) by downloading the relevant proteomes from UniProt [31, 32]. We combined the genome of *Mus musculus* (UP000000589) with genomes of the strains used in this study (Table 1) and genomes to represent the origins of dietary proteins: *Gallus gallus* (Chicken UP000000539—egg white protein)*, Glycine max* (Soybean UP000008827—soy protein and soybean oil)*, Bos taurus* (Cow UP000009136—casein)*, Zea mays* (Corn—corn fiber, corn oil, cornstarch)*, Helianthus annuus* (Sunflower—sunflower oil)*, and Beta vulgaris* (Sugar beet—sucrose)*.* For the dietary and mouse proteomes, the protein sequences were clustered with an identity threshold of 95% using CD-HIT [33]. The protein sequences of the bacterial strains were not clustered. Also included in the database were sequences of common laboratory contaminants (http://www.thegpm.org/crap/). The database contains a total of 324,982 protein sequences and is available from the data submitted to the PRIDE repository.

Protein identification and quantification.

For peptide and protein identification, MS data were searched against the protein database using the Sequest HT node in Proteome Discoverer version 2.3.0.523 (ThermoFisher Scientific) with the following parameters: digestion with trypsin (Full), maximum of two missed cleavages, 10 ppm precursor mass tolerance, 0.1 Da fragment mass tolerance and maximum three equal dynamic modifications per peptide. We considered the following dynamic modifications: oxidation on M (+15.995 Da), carbamidomethyl on C (+57.021 Da), and acetyl on the protein N terminus (+42.011 Da). Peptide false discovery rate (FDR) was calculated using the Percolator node in Proteome Discoverer, and only peptides identified at a FDR <5% were retained for protein identification. Proteins were inferred from peptide identifications using the Protein-FDR Validator node in Proteome Discoverer with a target FDR of 5%. We generated files of individual samples by combining the four replicate LC–MS/MS-produced files in the search. We used the resulting peptide-spectrum match (PSM) file for the protein-SIF method.

### Protein stable isotope fingerprinting

We used the Calis-p 2.0 software [21] to determine stable isotopic fingerprints of the organisms in the samples. The Calis-p software requires two input files: raw spectral files produced by the LC–MS/MS, and the PSM files containing the protein identifications and quantifications. Raw files were converted to mzML format using the MSConvertGUI tool via ProteoWizard [34] with the following options: Binary encoding precision: 64-bit, Write index: checked, TPP compatibility: checked, Filter: Peak Picking, Algorithm: Vendor, MS Levels: 1. The PSM files were generated as described above, and input into the Calis-p software as tab-delimited text files.

Calis-p performs two main steps: isotopic pattern extraction and SIF computation. The software first filtered out “ambiguous” peptides of low identification confidence from the input PSM file. Then, for each remaining peptide identification, the software found the corresponding mass spectrum in the mzML and extracted the isotopic pattern. After some clean-up and filtering steps, the software compared the experimentally derived isotopic pattern to *in silico* derived isotopic patterns to infer δ^13^C values for the peptide. This step was repeated for all peptides. Finally, the intensity-weighted average δ^13^C of the peptides from an organism was used to estimate that organism’s signature. We filtered the results to retain only SIF values computed from at least 30 peptides, which is the threshold required by the Calis-p 2.0 software to accurately estimate an organism’s SIF.

We corrected for the offset introduced by the mass spectrometer using both the human hair standard and the casein/egg standard by collecting δ^13^C values for the standards obtained both by protein-SIF and EA-IRMS and calculating the offset between the two methods [20]. We used the averaged offset value to correct the protein-SIF values of the organisms in the microbiome samples.

### Data analyses

We compared the SIF of each organism (Table 1) to the signatures of the dietary components fed to the mice. We looked for correspondence between signatures of organisms and signatures of dietary components to hypothesize which dietary constituents were assimilated by each organism. We also looked at how each organism’s SIF changed over time due to the different diet inputs to inform further data analyses. We identified significant differences (*P* < .05) using pairwise t-tests corrected for multiple hypotheses testing (Benjamini-Hochberg correction), computed in R version 4.0.2. We included the SIF values of the standard chow samples in the results to assess reproducibility. However, we did not know the signatures of the dietary constituents of the standard chow diet, and thus we did not test for significant differences between the standard chow and the defined diets. We prepared plots for organisms that had at least 3 data points from each of a minimum of two defined diets so that we could perform statistical analyses.

To estimate abundances of the different species, we applied the protein biomass method we developed previously [35]. Briefly, we filtered the identified proteins to proteins that had at least 2 protein unique peptides (2PUP proteins), we then summed the PSMs of all of the 2PUP proteins for each organism, and then calculated a percent proteinaceous biomass for each organism. We tested for significant changes in relative abundances using a one-way ANOVA followed by a Tukey's honestly significant difference (HSD) post hoc test, computed in R (version 4.0.2). We assigned letters to group means that are similar using the “rcompanion” package (version 2.4.1).

As a case study, to determine if the proteome of specific species could support the nutrient source hypotheses inferred from the protein-SIF data we calculated normalized protein abundance values (orgNSAF) for the proteins of *A. muciniphila*, *B. thetaiotaomicron*, and *M. formatexigens*. We then tested for significant differences in protein abundance between the different diets using an FDR-corrected ANOVA (q < 0.05). Plots were prepared in Origin 2018b, R pheatmap package, or in the Perseus software platform (version 1.6.12.0) [36], and compiled in Adobe Illustrator 2021.

## Results and discussion

### Experiment 1: changes in source of dietary protein impact isotopic signatures of intestinal microorganisms indicating their main carbon source.

**Figure 2:**
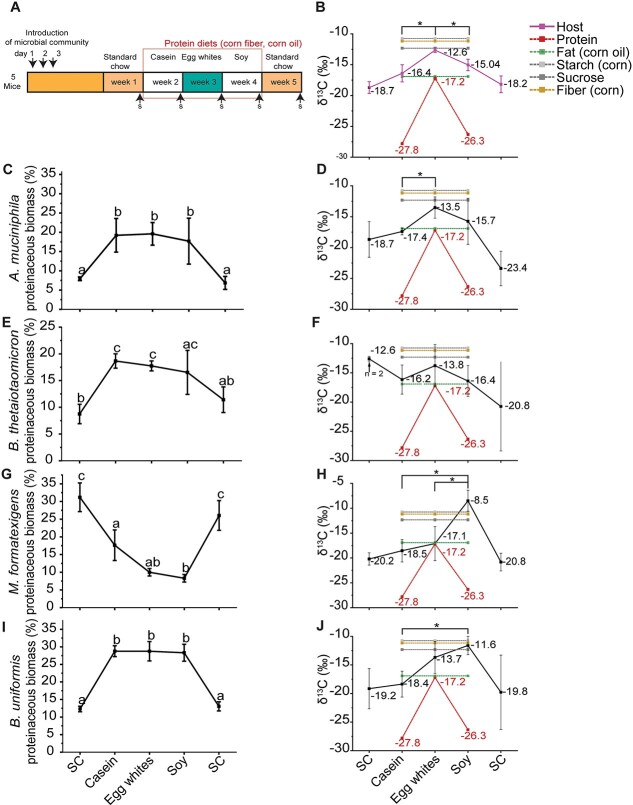
Changes in isotopic signatures of gut microorganisms in response to changes in the isotopic signature or source of dietary protein. (A) Overview of Experiment 1 as described in Materials & Methods (Fig. 1). (B, D, F, H, J) Protein-SIF δ^13^C values for mouse, *A. muciniphila*, *B. thetaiotaomicron*, *M. formatexigens* and *B. uniformis*. Displayed δ^13^C values for dietary protein sources, corn oil, corn starch, sucrose, and corn fiber were measured by isotope ratio mass spectrometry (IRMS). Significance is denoted by * and determined by T-tests corrected by BH (q < 0.05; n = 5 unless otherwise stated). (C, E, G, I) Relative proteinaceous biomass of *A. muciniphila*, *B. thetaiotaomicron*, *M. formatexigens* and *B. uniformis* determined according to the method described by [35]. Letters that do not overlap denote significantly different groups as determined by Tukey HSD (*P* < .05).

We fed gnotobiotic mice, colonized with a 13 member community (Table 1), a sequence of three defined diets that differed in their dietary protein source (casein, egg white, soy); our goal was to determine if these changes in dietary protein source affected the natural isotopic signature of intestinal microorganisms and the host, which would provide evidence for carbon source preferences. Isotope discrimination in heterotrophs is minor, usually between 0.5–2‰ [37, 38] per trophic level, whereas the marginal error rate of Protein-SIF is around 3‰ [20]. The δ^13^C values of the dietary protein sources were − 26.6‰ for casein, −17.2‰ for egg white, and − 26.3‰ for soy (Suppl. Table S2), which falls outside Protein-SIF’s margin of error. The isotopic signatures of all other dietary components were kept steady at −10.7‰ (cornstarch), −12.3‰ (sucrose), −11.2‰ (corn fiber), and − 16.9‰ (corn oil). We switched the protein source every seven days after collecting fecal samples (Fig. 2A). Our rationale was that a significant increase in the δ^13^C value of an organism between the casein and egg white diets followed by a decrease in δ^13^C value between the egg white and soy diets, i.e. a change in microbial δ^13^C value that tracked the diet’s change in δ^13^C value, would indicate that the organism uses dietary protein as a carbon source to make protein. We would not necessarily expect that the δ^13^C value of an organism becomes identical, or nearly identical, to that of a dietary component as an organism may use multiple substrates and carbon previously present in a cell will “dilute” the δ^13^C value of a newly used substrate. From each fecal sample we used metaproteomics to (i) extract microbial and host protein isotopic signatures using the protein-SIF approach [20]; and (ii) determine microbial community composition in terms of proteinaceous biomass [35].

The protein-SIF signature of host proteins detected in the feces increased significantly in the egg white protein diet compared to the other two diets: the average mouse δ^13^C value on egg white was −12.6‰, compared to −16.4‰ and − 15.0‰ on casein and soy protein, respectively, (BH corrected t-test *q* < 0.05) (Fig. 2B). Because the change in δ^13^C value of the host proteins mirrors that of the diet, this indicates that dietary protein is a carbon source for the host.

We were able to determine δ^13^C values for four members of the defined community: *A. muciniphila*, *B. thetaiotaomicron*, *M. formatexigens,* and *B. uniformis*. The abundance of *A. muciniphila* significantly (Tukey HSD *P* < .05) increased when switching from the initial standard chow diet to the casein diet and did not change after the casein-to-egg white or egg white-to-soy switches (Fig. 2C). The δ^13^C value of *A. muciniphila* significantly increased, to −13.5‰, in response to the egg white diet, indicating *A. muciniphila*’s metabolism responded to changes in dietary protein, mirroring the isotopic signature changes in the host (Fig. 2D). Because the host also responds to the dietary protein source in the same way and *A. muciniphila* is known to grow on host intestinal glycoproteins (mucins) [13, 39], this result is likely due to the use of host proteins as a carbon source by *A. muciniphila*. The abundance of *B. thetaiotaomicron* also increased after transitioning from standard chow to the casein diet and remained at a similar level with the egg white and soy diets (Fig. 2E). Although the δ^13^C value of *B. thetaiotaomicron* was higher under the egg white than the casein diet, this difference and other δ^13^C value comparisons were not statistically significant (Fig. 2F). The δ^13^C values of *M. formatexigens* and *B. uniformis* were the highest under the soy protein diet, with some differences being statistically significant (Fig. 2H and 2J). This was unexpected, because the δ^13^C value of soy protein is lower than the preceding egg white protein and therefore the expectation was that the δ^13^C values of these species would decrease if they were using protein as a carbon source or remain stable if not using protein as a carbon source. Instead, the increasing δ^13^C values suggest that *M. formatexigens* and *B. uniformis* transitioned from incorporating carbon from dietary or host protein to incorporating most carbon from the available carbohydrate sources (e.g. starch, sucrose, or corn fiber) when the diet was transitioned from casein to soy protein. The abundance of *M. formatexigens* decreased between the casein and soy protein diets (Fig. 2G), whereas the abundance of *B. uniformis* did not change in response to dietary protein source (Fig. 2I). Together these results indicate that the isotopic signatures of microbiota members and the host respond within a maximum of seven days after changing the diet. The results also indicate that changes in abundance are not necessarily driven by continued consumption of the same major substrate (i.e. protein), and that when the available substrate changes, e.g. from egg white protein to soy protein, the bacteria may preferentially consume fiber or fat.

In addition to these bacteria for which we could get an isotopic signature, we were also able to measure the abundances of *B. ovatus, R. intestinalis, E. rectale, E. coli, B. caccae, B. intestinihominis, C. aerofaciens, and C. symbiosum,* but not for *Faecalibacterium prausnitzii* which was below the limit of detection (Suppl. Fig. S1)*. B. ovatus, B. intestinihominis,* and *E. rectale* significantly changed in abundance when transitioning between standard chow and the defined diets. *B. ovatus*, *E. coli*, *B. caccae*, *B. intestinihominis,* and *C. aerofaciens* significantly changed in abundance when transitioning between sources of dietary protein. Together these results show that dietary protein source alters the abundances of specific bacteria in the gut as we have previously shown in conventional mice [27].

### Experiment 2: changes in source of fiber or fat alters the isotopic signatures of *B. thetaiotaomicron*, *M. formatexigens,* and *A. muciniphila*

**Figure 3:**
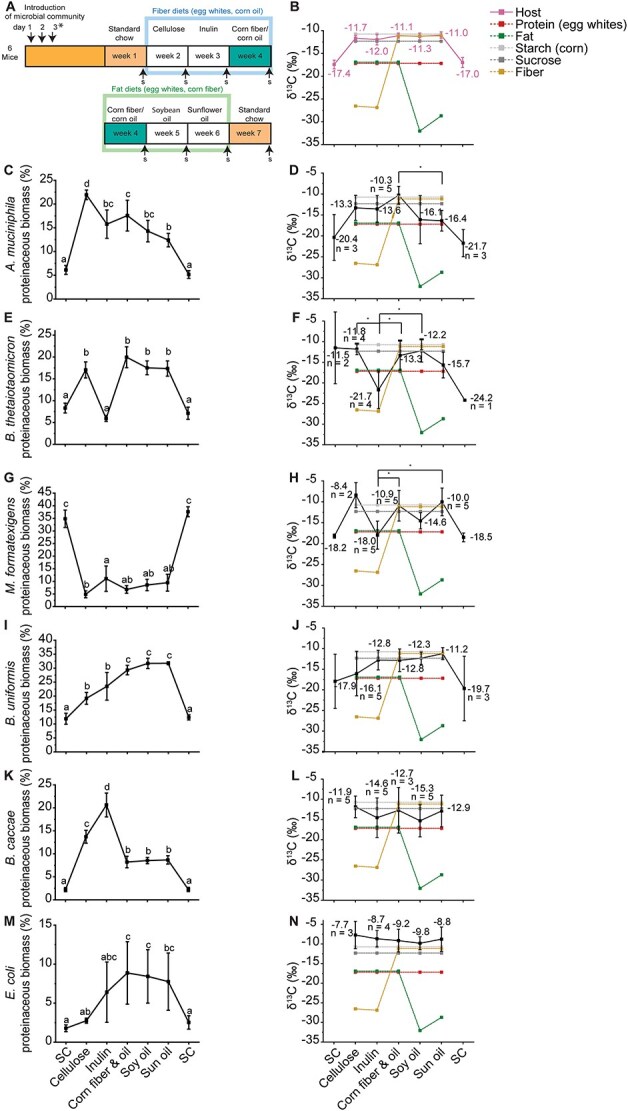
Changes in isotopic signatures of gut microorganisms in response to changes in the isotopic signature or source of dietary fiber or fat. A. Overview of Experiment 2 as described in Materials and methods (Fig. 1). (B, D, F, H, J, L, N) Protein-SIF δ^13^C values for mouse, *A. muciniphila*, *B. thetaiotaomicron*, *M. formatexigens*, *B. uniformis*, *B. caccae*, and *E. coli*, respectively. Displayed δ^13^C values for dietary fiber and fat sources, corn starch, sucrose, and egg white protein were measured by isotope ratio mass spectrometry (IRMS). Significance is denoted by * and determined by T-tests corrected by BH (q < 0.05; n = 6 unless otherwise stated). (C, E, G, I, K, M) Relative proteinaceous biomass of *A. muciniphila, B. thetaiotaomicron, M. formatexigens and B. uniformis, B. caccae,* and *E. coli*. Letters that do not overlap denote significantly different groups as determined by Tukey HSD (*P* < .05).

For experiment 2, we colonized germ-free mice with the same gnotobiotic community, but offered only egg white as the source of dietary protein. We first changed the fiber source each week, going from cellulose (−26.5‰), to inulin (−26.9‰), to corn fiber (−11.2‰) (Fig. 1A&B; Fig. 3A). We then kept the fiber isotopic signature constant by using corn fiber and changed the fat source each week from corn oil (−16.9‰), to soybean oil (−32.1‰), and to sunflower oil (−28.7‰). Neither fiber nor fat source significantly altered the δ^13^C value of the mouse proteins, which hovered at approximately −11‰ (Fig. 3B).

The only microorganism whose isotopic signature changed in response to fat source was *A. muciniphila*. Its δ^13^C value decreased steadily as it transitioned between the corn oil, soybean oil, and sunflower oil diets with a significant difference between the δ^13^C values under the corn oil diet and the sunflower oil diet (Fig. 3D). Both soybean oil and sunflower oil have much lower δ^13^C values than corn oil. The abundance of *A. muciniphila* was also responsive to fiber and fat source (Fig. 3C). Its abundance significantly increased from the standard chow to the defined diets, reaching its highest level under the cellulose diet. Additionally, the abundance of *A. muciniphila* significantly decreased in the sunflower oil diet relative to the cellulose, inulin, and corn fiber-corn oil diets.

Both *B. thetaiotaomicron* and *M. formatexigens* changed significantly in their abundances and isotopic signatures in response to changes in source of fiber. The abundance and isotopic signature of *B. thetaiotaomicron* significantly decreased when switching the diet from cellulose to inulin (Fig. 3E & F). This was unexpected because cellulose and inulin had similar δ^13^C values and thus the change in *B. thetaiotaomicron* isotope signature cannot be explained by continued use of fiber as carbon substrate. The δ^13^C values of other potential substrates for *B. thetaiotaomicron* including starch, sucrose, and the host were much higher than the ones of cellulose and inulin. Taken together this suggests that the change in *B. thetaiotaomicron* isotopic signature was driven by a switch from one of these nutrients to inulin despite inulin not leading to an increase in the abundance of *B. thetaiotaomicron.* In the case of *M. formatexigens*, the bacterium had a lower δ^13^C value in the inulin diet relative to the corn fiber containing diets and trended towards a lower δ^13^C in the inulin diet relative to the cellulose diet (Fig. 3F). In this case, *M. formatexigens* significantly increased in abundance between cellulose and inulin suggesting that the transition towards incorporating inulin in this case increased the abundance of *M. formatexigens* (Fig. 3E).

Isotopic signatures for *B. uniformis*, *B. caccae*, and *E. coli* did not change in response to changes in fiber or fat sources (Fig. 3J, 3L, and  3N). All three of these bacteria, however, changed in abundance due to fiber source. *B. uniformis* (Fig. 3I) significantly increased in abundance between the cellulose and corn fiber-corn oil diets, *B. caccae* significantly increased in abundance between cellulose and inulin then significantly decreased in the presence of corn fiber (Fig. 3K), and *E. coli* increased in abundance between the cellulose and corn fiber diets as well (Fig. 3M).

We were able to calculate the abundances of *B. ovatus*, *R. intestinalis*, *E. rectale*, *B. intestinihominis*, *C. symbiosum*, and *C. aerofaciens,* but not for *F. prausnitzii* which was below the limit of detection (Suppl. Fig. S2). None of these bacteria had a significant change in abundance due to fat source, but they all experienced significant changes in abundance due to fiber source suggesting that fiber source has a much greater impact on gut microbiota composition than fat source.

### Differential metaproteomic analysis reveals additional information about carbon source choices of specific species

We analyzed the metaproteomic data to identify gene expression changes of specific species in response to diet changes to gain additional insights into the nutrient/carbon sources of species with unexpected changes in their isotopic signatures. We focused on *M. formatexigens* in Experiment 1 and the fiber component of Experiment 2, *B. thetaiotaomicron* in the fiber component of Experiment 2, and *A. muciniphila* in Experiment 1 and in the fat component of Experiment 2.

**Figure 4:**
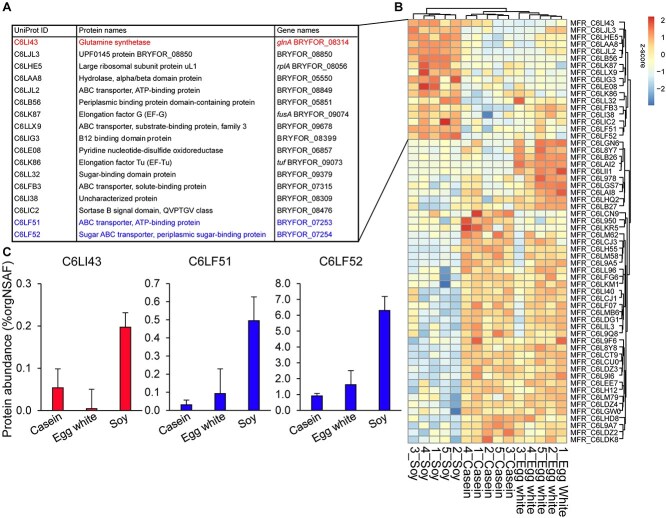
Hierarchical clustering of *M. formatexigens* proteins that significantly differed between casein, egg, white, and soy protein. (A) Table listing 17 proteins that were more abundant in the soy cluster. (B) Hierarchical clustering of the z-score values of the 60 proteins that changed significantly in abundance between the three dietary protein sources (ANOVA, q < 0.05, n = 5). (C) Abundant proteins that potentially explain the increase in the δ^13^C value in the soy diet.(Fig. 2H). Glutamine synthetase is highlighted in red; proteins from a Sugar ABC transporter gene neighborhood are highlighted in blue. Bars represent average %orgNSAF abundance and error bars represent the standard deviation.

We identified 60 *M. formatexigens* proteins that significantly changed in abundance between casein, egg white, or soy protein (ANOVA, q < 0.05). Hierarchical clustering of samples using these proteins revealed 17 proteins that were more abundant in the soy diet relative to the other diets (Fig. 4). Among these were two proteins that are components of a sugar ABC transporter, which suggests more sugar was being imported under the soy diet. Also increased in the soy diet was the abundance of *M. formatexigens’* glutamine synthetase, which is a protein that is upregulated in bacteria under nitrogen limitation because it plays a major role in the incorporation of inorganic nitrogen [40]. Together these results suggest that the soy diet-induced shift in *M. formatexigens’* isotopic signature was due to a switch from protein to sugar as the primary carbon source. It is unclear if the increase in glutamine synthetase expression was caused by generally lower nitrogen availability in the gut during the soy protein diet or by a need for *M. formatexigens* to acquire inorganic nitrogen for de novo amino acid synthesis when not using soy protein as a source of organic carbon and nitrogen.

In the fiber component of Experiment 2, *M. formatexigens* differentially expressed 133 proteins between the three different fiber sources (ANOVA, q < 0.05). Among these 38 were significantly increased in the inulin diet including a protein from the CAZy family GH32 (UniProt ID C6LAL4), which is described as containing inulin and levan fructosidases in the CAZy database [41]. This supports the suggestion that *M. formatexigens* is incorporating carbon from inulin due to the decrease in δ^13^C when transitioning to that diet. This also makes sense because it was previously shown that *M. formatexigens* can grow on inulin [13].

**Figure 5:**
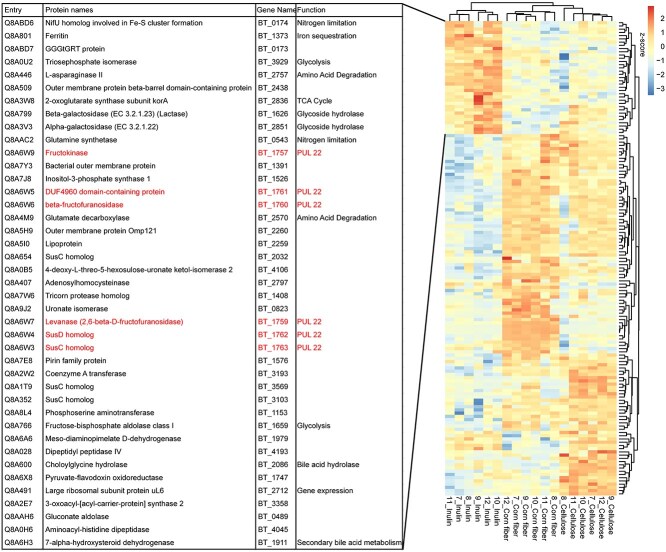
Hierarchical clustering of *B. thetaiotaomicron* proteins that significantly differed between cellulose, inulin, and corn fiber. Hierarchical clustering of the z-score values of the 147 proteins whose abundances changed significantly between the three dietary fiber sources (ANOVA, q < 0.05, n = 5). The table represents the 41 proteins abundant in the inulin cluster. Proteins from PUL22 are highlighted in red.

**Figure 6:**
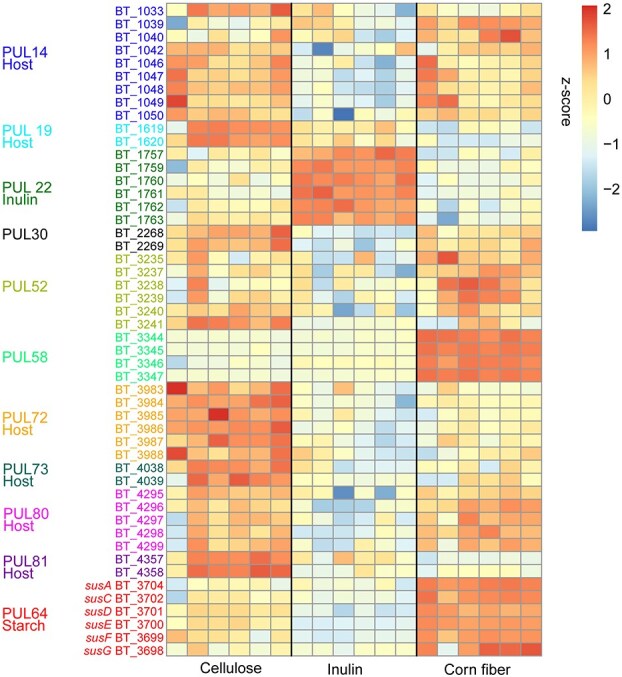
*B. thetaiotaomicron* proteins from a PUL that significantly differed in abundance between the diets with different fiber sources. Heatmap ordered by diet (cellulose, inulin, corn fiber) representing the z-scored abundances of 50 proteins from PULs ordered by the PULs. If the substrate of the PUL is known, it is described. All PUL numbers are from the literature-derived numbering in PULDB.

We identified 147 *B. thetaiotaomicron* proteins whose abundances significantly differed between the cellulose, inulin, and corn fiber diets (ANOVA, q < 0.05). Using hierarchical clustering, these proteins separated into distinct inulin, cellulose, and corn fiber clusters. The inulin cluster was most distinct containing 41 proteins whose abundances increased in response to inulin relative to the other diets (Fig. 5). Fifty of the 147 significantly different proteins, approximately ⅓, belong to polysaccharide utilization loci (PUL) [42, 43] (Fig. 6). PULs are *Bacteroides* gene neighborhoods that encode all the enzymes needed to import and degrade a specific glycan structure. Six of these PUL-derived proteins, which were among the 41 proteins significantly increased under the inulin diet relative to the other diets, represent ⅔ of the nine proteins encoded by the inulin- and levan-degrading PUL22 (All PUL numbers are from the lit-derived numbering in PULDB) [43, 44]. The remaining PULs were elevated in cellulose or corn fiber relative to inulin. PUL66 is a starch degrading PUL, although PUL14, PUL19, PUL72, PUL73 PUL80, and PUL81 have been previously linked to degrading host protein glycosylations [15]. We also recently linked PUL14, PUL72, and PUL80 to the degradation of egg white protein glycosylations, which have glycan structures similar to host intestinal mucin [27]. Together with the observed changes in δ^13^C values under the inulin diet (Fig. 3F), these results suggest that *B. thetaiotaomicron* transitioned from using starch and a combination of host and dietary proteins under the cellulose and corn fiber diets, to using inulin as its primary carbon source under the inulin diet.

Because *B. thetaiotaomicron’s* abundance decreased under the inulin diet, its switch to inulin utilization is puzzling, given that its alternative substrates starch, host protein, and egg white dietary protein (which it used in the cellulose and corn fiber diets) were included in the inulin diet at the same amount as in the cellulose and corn fiber diets. Previous research showed that *B. thetaiotaomicron* does not grow well on inulin and that the upregulated PUL22 enables more efficient degradation of the fructan levan as compared to inulin [44]. Taken together this leads us to speculate that the presence of a fructan (levan or inulin) induces expression of PUL22 and downregulates genes for the use of other carbon sources in *B. thetaiotaomicron*, whether this is beneficial for growth or not.

We also inspected the proteome of *A. muciniphila* in both Experiment 1 and the fat component of Experiment 2 because *A. muciniphila* responded with changes in δ^13^C value to changes in both dietary protein and fat. The abundances of only nine proteins were significantly different in Experiment 1 (ANOVA, q < 0.05) and no proteins significantly differed between fat sources in Experiment 2 (ANOVA, q < 0.05), implying diet had minimal impact on *A. muciniphila’s* metabolism despite the changes in its isotopic signature in both experiments. *A. muciniphila* is known to grow almost exclusively on mucin derived from the host [13, 39] or similarly glycosylated proteins and the proteome supported this because we detected all the proteins needed to metabolize common mucin components like fucose, acetylglucosamine, sialic acid, and degradation enzymes like sialidases and beta-N-acetylhexosaminidases (Suppl. Table S3). Therefore “foraging” of host mucin could explain the changes in *A. muciniphila’s* δ^13^C value in Experiment 1. The change in *A. muciniphila’s* δ^13^C value could, however, also be at least in part due to direct use of egg white protein as a substrate. This notion is supported by a recent study, in which the abundance of *A. muciniphila* increased when egg white was provided as the dietary protein source [27], and another study which showed that *A. muciniphila* grows on specific egg white proteins [45]. In Experiment 2, the δ^13^C value of host proteins was unchanged despite changes in fiber and fat source, yet *A. muciniphila’s* δ^13^C value significantly decreased between corn oil and sunflower oil. This change in the δ^13^C value corresponded with no change in the proteome of *A. muciniphila*.

We speculate that *A. muciniphila* consistently consumes mucin under any diet, and that different components of mucin have different isotopic signatures. Mucins are heavily glycosylated proteins, and carbon for glycosylations can come from different dietary components than carbon for the amino acids used to generate the protein backbone, which is the basis for the isotopic signature detected using protein-SIF. Specifically, sugars for glycosylations can come in part from conversion of carbohydrates and in part from gluconeogenesis with fatty acids as precursors [46]. This would lead to the δ^13^C value of glycosylations to change in response to changes in the isotopic signature of fiber and fat sources, even though the δ^13^C value of the mucin protein backbone remains unchanged as the amino acids are obtained by the host from dietary protein. Because *A. muciniphila* is predicted to be able to synthesize all the amino acids except threonine [47], it is possible that it uses carbon from mucin glycan conjugates for amino acid biosynthesis. Thus, changes in the isotopic signature of mucin glycosylations become evident in the protein δ^13^C value of *A. muciniphila*. This is consistent with evidence suggesting that fat source affects glycosylations of intestinal mucins [48].

## Conclusion

We used the protein-SIF approach to track the *in vivo* utilization of specific protein, fiber, and fat substrates by several species of intestinal bacteria, and quantified how utilization of these carbon sources affected accumulation of proteinaceous biomass for each bacterial species. We found that the abundance of particular species is not a good indicator of the species’ preference for a specific dietary component. Moreover, using the same metaproteomics data, we identified changes in the expression of genes that underlie the use of specific carbon sources showing that metaproteomics gene expression data can be used in tandem with the protein-SIF data to make stronger inferences about the carbon substrates of specific species. Ultimately, we were able to capture seven separate instances where a change in protein, fiber, or fat source correlated with a significant change in the protein δ^13^C value of a specific organism including the host, providing direct evidence for intestinal microorganisms using particular dietary components as carbon sources. Additionally, we found that some microbial species switch between macronutrients (protein, fiber or fat) when the source of their previously preferred macronutrient changes (Table 2).

**Table 2:** Description of significant changes in δ^13^C value and their interpretation.

Species	δ^13^C value switch	Figure	Interpretation
*M. musculus* (host)	Significant increase in δ^13^C value under egg white diet relative to casein and soy protein diets.	Figure 2B	Host uses dietary protein as a carbon source for synthesizing protein.
*Akkermansia muciniphila*	Significant increase in δ^13^C value under egg white diet relative to casein and soy protein diets.	Figure 2D	Forages host protein (mucin) and/or dietary protein, thus following the isotopic signature of the dietary protein source.
*Akkermansia muciniphila*	Significant decrease in δ^13^C value under sunflower oil diet relative to corn oil diet.	Figure 3D	Continues using host mucin (glycosylated protein) as carbon source, but we speculate that the δ^13^C of the glycosylations has decreased due to the low δ^13^C value of sunflower oil.
*Marvinbryantia formatexigens*	Significant increase in δ^13^C value under soy relative to egg white and casein protein diets.	Figure 2H	Switches from dietary protein to sugar as primary carbon source in the soy protein diet.
*Bacteroides uniformis*	Significant increase in δ^13^C value under soy relative to casein protein diet.	Figure 2J	Switches from dietary protein to sugar as primary carbon source in the soy protein diet.
*Bacteroides thetaiotaomicron*	Significant decrease in δ^13^C value under inulin diet relative to cellulose and corn fiber.	Figure 3F	Switches from starch, dietary protein and/or host proteins to inulin as primary carbon source.
*Marvinbryantia formatexigens*	Significant decrease in δ^13^C value under inulin diet relative to corn fiber.	Figure 3H	Switches from starch, dietary, or host proteins to inulin as primary carbon source.

Our study has several limitations that could be addressed in future work. First, due to the current detection limits of the protein-SIF approach we were only able to measure δ^13^C values for the more abundant members of the 13 species in the community. Currently, the speed of data acquisition of mass spectrometers is rapidly advancing and will enable calculation of δ^13^C values from more community members in future experiments. We anticipate that with these advances, protein-SIF will be a powerful tool for investigating the ecology of dietary nutrient usage and niche differentiation in a variety of different host-microorganism systems. Second, in this study we used gnotobiotic mice with a defined community, which has the advantage that we can use the exact protein sequences of all microbiota members to identify proteins, but it limits our understanding of how carbon sources are used in more complex, natural microbiota. We do not foresee any obstacles to applying protein-SIF to natural intestinal microbiota, particularly for the more abundant species. Third, we used fully defined diets, which are advantageous because we can precisely measure and control isotopic signatures of each diet, and attribute changes in isotopic signature of microbial species to a specific source of the dietary component. However, fully defined diets are limited in that observed diet-microorganism interactions may be driven by the “artificial”, low complexity nature of the diets. For example the unexpected switch of *B. thetaiotaomicron* to inulin utilization, which coincided with a reduction in its biomass, may not represent a realistic scenario, as the bacteria was only provided with inulin, whereas dietary fiber is usually present as a complex mixture of fiber types.

The flexibility to freely manipulate diet and then use protein-SIF has the potential to be applied quite broadly. For example, protein-SIF could be applied in the context of controlled feeding studies in humans, [8, 49], with limited safety concerns because there is no need to artificially label substrates. This could e.g. be done by feeding people controlled diets where the baseline diet consists of products from C4 plants and then a single animal protein or C3 plant protein source is included. The δ^13^C values of C4 plant-based foods are distinct from those of C3 plants and certain animals. The animal protein source or C3 plant source could then be changed and we would be able to observe changes in the δ^13^C values of specific microorganisms. Protein-SIF should work in more complex human microbiomes as we have already successfully assigned proteins to specific microorganisms in mice with a conventional microbiota, which is similar in complexity to the microbiota of humans [27, 50]. Paired with the gene expression information from metaproteomics we anticipate that Protein-SIF will be a powerful tool for understanding substrate utilization in human microbiota in future studies.

## Supplementary Material

Supplementary_Materials_wraf127

## Data Availability

The mass spectrometry metaproteomics data and protein sequence database were deposited to the ProteomeXchange Consortium via the PRIDE [51] partner repository. PXD046928.
