# Supplementary material for: Metaproteomics-based stable isotope fingerprinting links intestinal bacteria to their carbon source and captures diet-induced substrate switching
Source: ISME J. 2025 Jun 24;19(1):wraf127. doi: 10.1093/ismejo/wraf127 (PMC12306441; doi:10.1093/ismejo/wraf127)
Supplement: Supplementary_Materials_wraf127 [file supplementary_materials_wraf127.pdf]

Metaproteomics-based stable isotope fingerprinting links intestinal bacteria to their carbon source and captures diet-induced substrate switching

Angie Mordant[1]†, J. Alfredo Blakeley-Ruiz[1]†, Manuel Kleiner[1]

1: Department of Plant and Microbial Biology, North Carolina State University, Raleigh NC

Corresponding Author Information
Manuel Kleiner - manuel_kleiner@ncsu.edu

† These authors contributed equally

Supplementary Materials

Supplementary Table S1: Diet formulations.

Teklad ID	TD.200545	TD.200546	TD.200547	TD.200548	TD.200549	TD.200550	TD.200551
Name	Egg white protein, corn oil, soluble corn fiber diet	Cellulose fiber diet	Inulin fiber diet	Sunflower oil diet	Casein protein diet	Soy protein diet	Soybean oil diet
Protein	Egg white 200g/Kg	Egg white 200g/Kg	Egg white 200g/Kg	Egg white 200g/Kg	Casein 200g/Kg	Soy 200g/Kg	Egg white 200g/Kg
Corn starch	351.371 g/Kg	351.371 g/Kg	351.371 g/Kg	351.371 g/Kg	351.371 g/Kg	351.371 g/Kg	351.371 g/Kg
Maltodextrin	132 g/Kg	132 g/Kg	132 g/Kg	132 g/Kg	132 g/Kg	132 g/Kg	132 g/Kg
Sucrose	100 g/Kg	100 g/Kg	100 g/Kg	100 g/Kg	100 g/Kg	100 g/Kg	100 g/Kg
oil	Corn oil 120 g/Kg	Corn oil 120 g/Kg	Corn oil 120 g/Kg	Sunflower oil 120 g/Kg	Corn oil 120 g/Kg	Corn oil 120 g/Kg	Soybean oil 120 g/Kg
fiber	Soluble corn fiber 50 g/Kg	Cellulose 50 g/Kg	Inulin 50 g/Kg	Soluble corn fiber 50 g/Kg	Soluble corn fiber 50 g/Kg	Soluble corn fiber 50 g/Kg	Soluble corn fiber 50 g/Kg
Mineral mix	13.39 g/Kg	13.39 g/Kg	13.39 g/Kg	13.39 g/Kg	13.39 g/Kg	13.39 g/Kg	13.39 g/Kg
Calcium phosphate	12.02 g/Kg	12.02 g/Kg	12.02 g/Kg	12.02 g/Kg	12.02 g/Kg	12.02 g/Kg	12.02 g/Kg
Calcium Carbonate	3.43 g/Kg	3.43 g/Kg	3.43 g/Kg	3.43 g/Kg	3.43 g/Kg	3.43 g/Kg	3.43 g/Kg
Vitamin mix	15 g/Kg	15 g/Kg	15 g/Kg	15 g/Kg	15 g/Kg	15 g/Kg	15 g/Kg
Choline	2.75 g/Kg	2.75 g/Kg	2.75 g/Kg	2.75 g/Kg	2.75 g/Kg	2.75 g/Kg	2.75 g/Kg
Biotin	0.003 g/Kg	0.003 g/Kg	0.003 g/Kg	0.003 g/Kg	0.003 g/Kg	0.003 g/Kg	0.003 g/Kg
Thiamin	0.01 g/Kg	0.01 g/Kg	0.01 g/Kg	0.01 g/Kg	0.01 g/Kg	0.01 g/Kg	0.01 g/Kg
Vitamin K	0.002 g/Kg	0.002 g/Kg	0.002 g/Kg	0.002 g/Kg	0.002 g/Kg	0.002 g/Kg	0.002 g/Kg
TBHQ	0.024 g/Kg	0.024 g/Kg	0.024 g/Kg	0.024 g/Kg	0.024 g/Kg	0.024 g/Kg	0.024 g/Kg

Supplementary Table S2. EA-IRMS results of dietary components used in this study. Unless indicated, all ingredient samples were obtained from Envigo Teklad. Duplicate dietary component rows indicate duplicate measurements.

Dietary component	mg C	mg N	δ^{13}C	δ^{15}N	C:N	Avg. δ^{13}C (n=2)	Diff. reps (n = 2)	Date tested
cornstarch	0.073		-10.84			**-10.75**	0.13	Jan 10 2019
cornstarch	0.122		-10.66					Jan 10 2019
sucrose	0.061		-12.18			**-12.32**	0.19	Jan 10 2019
sucrose	0.204		-12.46					Jan 10 2019
cellulose	0.193		-26.41			**-26.55**	0.20	Jan 10 2019
cellulose	0.144		-26.69					Jan 10 2019
inulin	0.125		-26.92			**-26.88**	0.06	Jan 10 2019
inulin	0.092		-26.83					Jan 10 2019
maltodextrin maltrin	0.238		-10.40			**-10.40**	0.01	Jan 10 2019
maltodextrin maltrin	0.172		-10.39					Jan 10 2019
maltodextrin lodex	0.166		-10.58			**-10.65**	0.09	Jan 10 2019
maltodextrin lodex	0.101		-10.71					Jan 10 2019
casein	0.091	0.02829	-26.46	6.02	3.7	**-26.56**	0.15	Jan 10 2019
casein	0.095	0.02832	-26.67	5.19	3.9			Jan 10 2019
soy protein	0.112	0.03342	-26.36	-0.22	3.9	**-26.33**	0.05	Jan 10 2019
soy protein	0.070	0.02152	-26.29	-0.06	3.8			Jan 10 2019
egg white solids	0.147	0.04316	-17.03	4.31	4.0	**-17.19**	0.22	Jan 10 2019
egg white solids	0.113	0.03324	-17.35	4.05	4.0			Jan 10 2019
soybean oil	0.137		-32.06			**-32.05**	0.01	Jan 10 2019
soybean oil	0.315		-32.05					Jan 10 2019
corn oil	0.142		-17.16			**-16.92**	0.35	Jan 10 2019
corn oil	0.240		-16.67					Jan 10 2019
Corn fiber Amazon B00NAD0IVU	0.091	0.0005	-11.11			**-11.17**	0.07	Apr 2019
Corn fiber Amazon B00NAD0IVU	0.180	0.0005	-11.15					Apr 2019
Corn fiber Amazon B00NAD0IVU	0.255	0.0005	-11.24					Apr 2019
Sunflower oil Amazon B0792GCNWV	0.148		-30.95			**-31.24**	0.42	Apr 2019
Sunflower oil Amazon B0792GCNWV	0.240		-31.54					Apr 2019

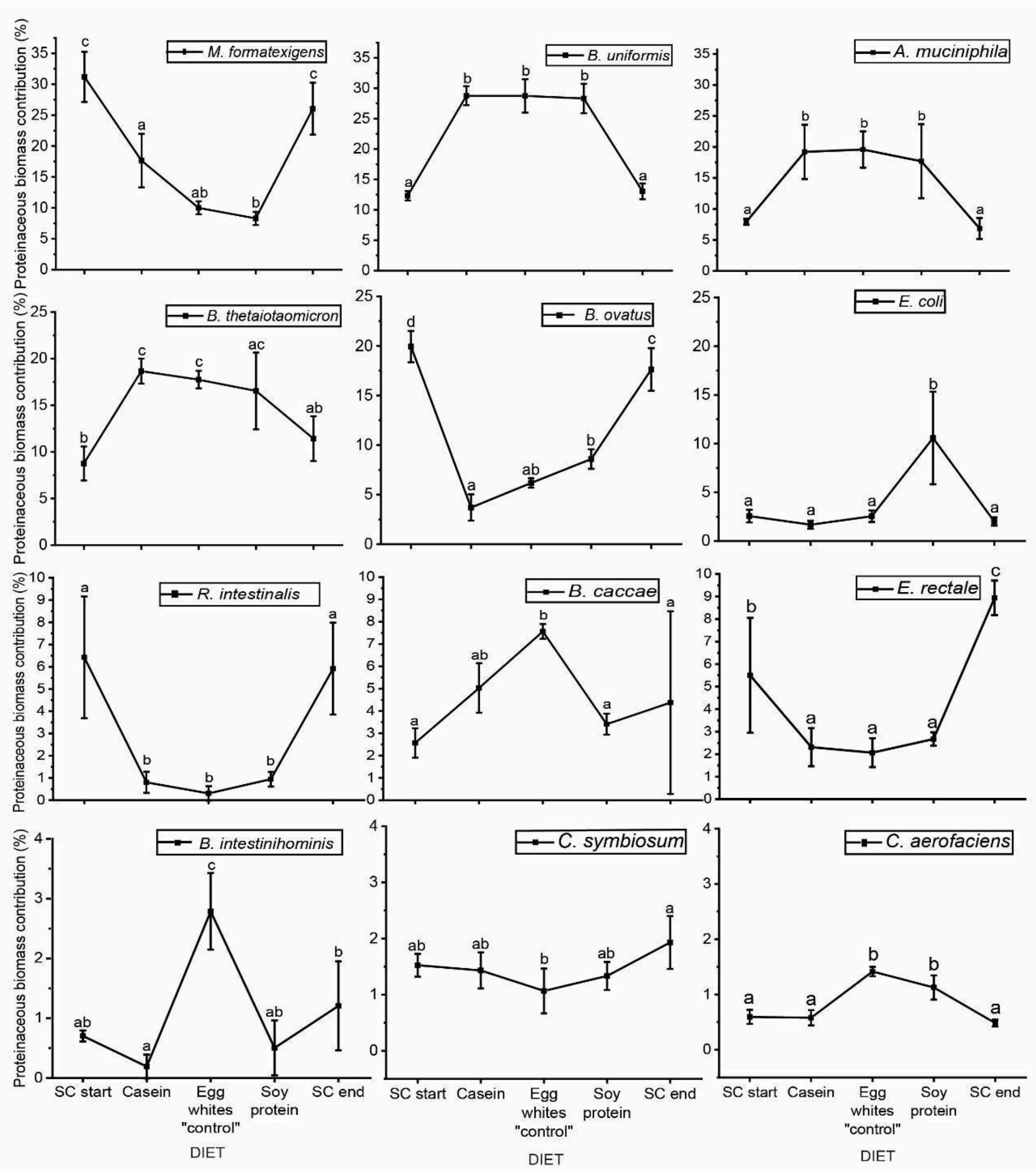

Supplementary Figure S1. Relative proteinaceous biomass contribution of *M. formatexigens, B. uniformis, A. muciniphila, B. thetaiotaomicron, B. ovatus, E. coli, R. intestinalis, B. caccae, E. rectale, B. intestinihominis, C. symbiosum, and C. aerofaciens* in mice fed the protein diets (Experiment 1). Each point represents the relative biomass contribution of the organism after the mice were fed the diet indicated on the x-axis for seven days. Relative abundances were averaged and error bars indicate standard deviation (n = 5). Diets are ordered on the x-axis in chronological order fed to the mice. SC = standard chow diet. Casein = diet with Casein as the protein source; Egg whites ("control") = diet with egg whites as the protein source; Soy protein = diet with soy protein as the protein source. Please note that the y-axis scale differs per row. Significant differences are indicated by different letters (a, b, c, d; based on one-way ANOVA and Tukey's HSD post hoc test, $P < .05$).

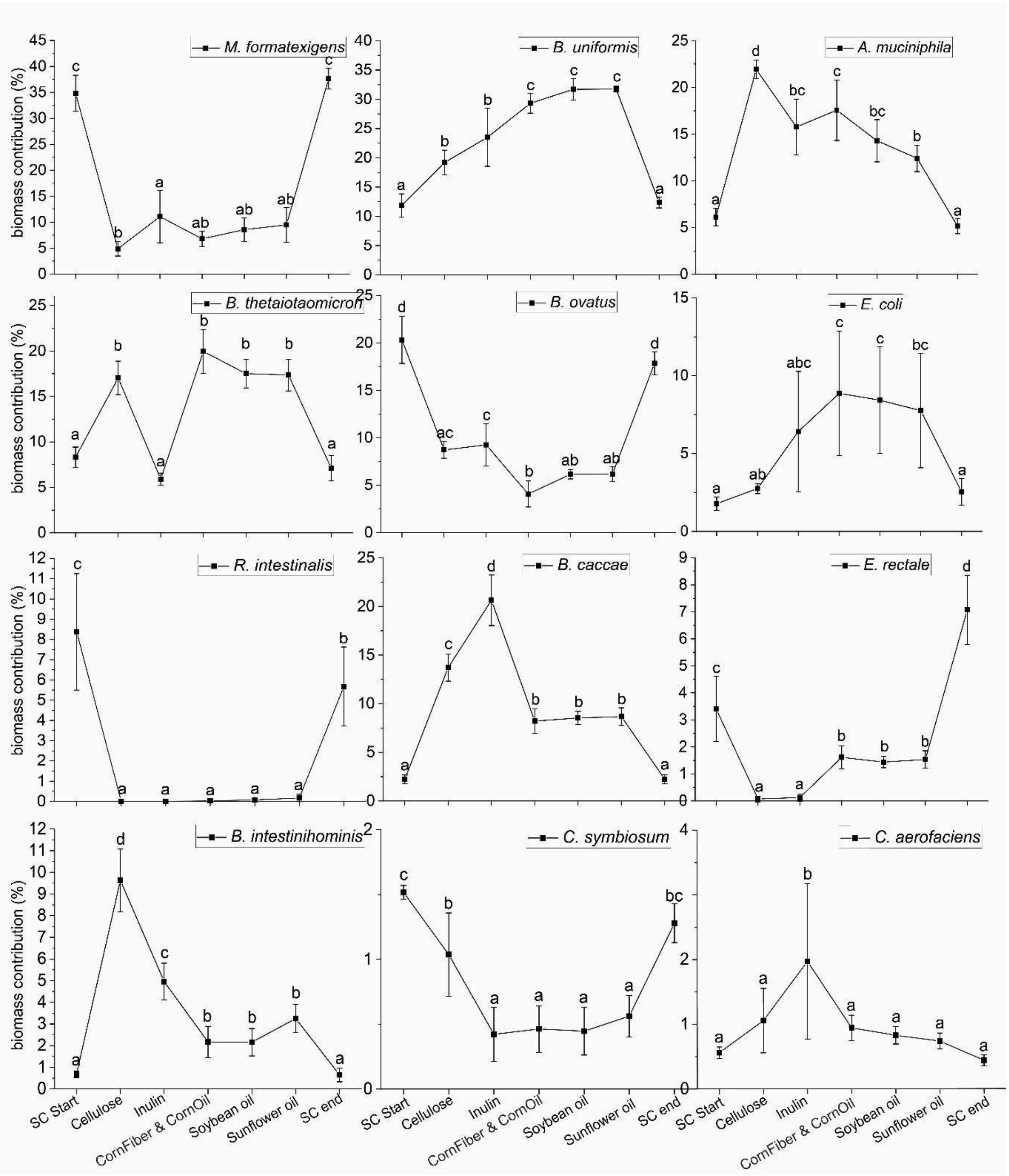

Supplementary Figure S2. Relative proteinaceous biomass contribution of *M. formatexigens, B. uniformis, A. muciniphila, B. thetaiotaomicron, B. ovatus, E. coli, R. intestinalis, B. caccae, E. rectale, B. intestinihominis, C. symbiosum*, and *C. aerofaciens* in mice fed the fiber and fat diets (Experiment 2). Each point represents the relative biomass contribution of the organism after the mice were fed the diet indicated on the x-axis for seven days. Relative abundances were averaged and error bars indicate standard deviation (n = 6). Diets are ordered on the x-axis in chronological order fed to the mice. SC = standard chow diet. Cellulose = diet with cellulose as the fiber source; Corn fiber & Corn oil ("control") = diet with corn fiber as the fiber source and corn oil as the fat source; Soybean oil = diet with soybean oil as the fat source. Sunflower oil = diet with sunflower oil as the fat source. Please note that the y-axis scale differs per row. Significant differences are indicated by different letters (a, b, c, d; based on one-way ANOVA and Tukey's HSD post hoc test, $P < .05$).

Supplementary Table S3. Mucin related *A. muciniphila* enzymes detected in proteome. Enzymes detected in the *A. muciniphila* proteome involved in the metabolism of mucin associated sugars sialic acid, fucose, and acetylglucosamine. The table includes the UniProt ID, the gene name, the protein name, relevant pathway, and it ranks abundance in the proteome with lower numbers being more abundant proteins.

UniProt ID	*A. muciniphila* gene name	Protein name	Mucin relevant pathway	Average Rank Abundance
B2UKW7	Amuc_1417	Glyceraldehyde-3-phosphate dehydrogenase	Glycolysis after fructose 6 phosphate	1
B2UN99	Amuc_372	Glutamate decarboxylase	Protein fermentation	17
B2UN29	Amuc_1822	Glucosamine-6-phosphate deaminase	Acetylglucosamine to fructose 6 phosphate	24
B2UKW8	Amuc_1418	Phosphoglycerate kinase	Glycolysis after fructose 6 phosphate	26
B2UP29	Amuc_0562	Triosephosphate isomerase	Glycolysis after fructose 6 phosphate	31
B2URF0	Amuc_1210	Phosphoenolpyruvate carboxykinase	Glycolysis after fructose 6 phosphate	33
B2UNP3	Amuc_1946	N-acetylneuraminate lyase	Sialic acid to acetylglucosamine	35
B2UQP5	Amuc_0948	N-acetylglucosamine-6-phosphate deacetylase	Acetylglucosamine to fructose 6 phosphate	39
B2URC4	Amuc_1184	Enolase	Glycolysis after fructose 6 phosphate	62
B2UQ23	Amuc_0721	fructose-bisphosphate aldolase, class II	Glycolysis after fructose 6 phosphate	64
B2UN39	Amuc_1832	L-fucose isomerase	Fucose to glyceraldehyde 3 phospfase	68
B2UNP4	Amuc_1947	N-acylglucosamine 2-epimerase	Sialic acid to acetylglucosamine	160
B2UPR7	Amuc_2136	Glycoside hydrolase, family 20	Releases acetylglucosamine residues from glycans	196
B2UN36	Amuc_1829	class II aldolase/adducin family protein	Fucose to glyceraldehyde 3 phospfase	341
B2UPI5	Amuc_0625	Exo-alpha-sialidase	Releases sialic acid residues from glycans	426
B2UN42	Amuc_1835	Exo-alpha-sialidase	Releases sialic acid residues from glycans	474
B2UQX3	Amuc_1032	Beta-N-acetylhexosaminidase	Releases acetylglucosamine residues from glycans	529
B2UN37	Amuc_1830	L-fuculokinase	Fucose to glyceraldehyde 3 phospfase	618
B2ULI1	Amuc_1547	Sialidas	Releases sialic acid residues from glycans	669
B2UP58	Amuc_2019	Beta-N-acetylhexosaminidase	Releases acetylglucosamine residues from glycans	767
B2UM43	Amuc_1669	Beta-N-acetylhexosaminidase	Releases acetylglucosamine residues from glycans	785
